# Surgery

**Published:** 1894-12

**Authors:** 


### SELECTIONS AND ABSTRACTS.

### SURGERY.

Edited by Floyd W. McRae, M.D.

### Appendicitis.

So much is being said now in all the journals about appendicitis, that we reproduce here an excellent digest of the most important recent literature (from the *Medical Chronicle*) on the subject:

Robson* shows how the appendix may be looked upon as a thick, hollow tube of adenoid tissue resembling the tonsils in structure, lined on its inner surface by a layer of epithelium, and covered on its outer by a muscular tube and a peritoneal investment. He points out how the continuity of the tube with the intestine, always containing septic material; the thin layer of epithelium alone guarding the tonsil-like tissue from infection; the narrow lumen of the tube, easily blocked by the swollen lymphoid tissue; the muscular layer, readily excited to severe and painful spasm; and the thin peritoneal investment, part of the general peritoneal cavity—constitute an anatomical series which may not inaptly be termed a veritable death-trap, when, from any local cause, whether in the shape of injury or disease, the protecting epithelium becomes displaced, allowing bacterial invasion of the lymphoid tissue, and producing an infective inflammation which is known as "appendicitis," and which may be acute, sub-acute or chronic.

Barling† points out that the appendicitis is so variable that it is absolutely necessary to classify the cases into separate groups. Taking a classification which is clinical, rather than pathological, he aims at grouping together cases with similar features and requiring similar treatment, and so divides them into the following classes: (1) Mild appendicitis; (2) appendicitis with distinct ab-

*Robson (Mayo). "On recurrent appendicitis, or recurring appendicular colic, with cases treated by operation in the quiescent period."—*Lancet*, June 30, 1894, p. 1608.

†Barling (Gilbert). "Appendicitis."—*Medical Annual*, 1894, p. 80.

scess formation; (3) acute perforating appendicitis; (4) relapsing appendicitis.

Lloyd* writes, "The more I see of typhlitic affections, the more firmly do I accept the theory that they begin primarily in the appendix, and that peritiphlitic lesions are secondary to them." He divides, for purposes of treatment, all typhlitic maladies into simple and complicated. The simple include "appendicular colic," simple parietal appendicitis, with or without localized fibrinous peritonitis, and certain cases of the sub-acute and recurrent varieties. The complicated include the acute perforative form with diffused peritonitis, or the less acute form with localized suppuration; some sub-acute, and probably all chronic "relasping" cases.

Senn† believes that appendicitis obliterans is a comparatively frequent form of relapsing appendicular inflammation. The oblitcrating process is of a progressive character, and may lead to complete destruction of all the glandular tissue and obliteration of the entire lumen. The changes begin either in the mucous coat and form superficial ulceration, or as an interstitial process following lymphatic infection. The symptoms are characterized by acute exacerbations of short duration and moderate intensity, but with no appreciable swelling at the seat of disease. Tenderness persists during the intermissions.

Edebohls‡ believes that the appendix may, under ordinary circumstances, be palpated; and states that "pressure deep enough to recognize distinctly the posterior abdominal wall, the pelvic brim, and the structures lying between them and the examining finger, forms the whole secret of success in the practice of palpation of the vermiform appendix."

Bull§ insists on the very great *variability* of the symptoms. An attack often begins with sudden pain, perhaps at first referred to the umbilical region, radiating then over the abdomen, and gradually becoming localized on the right iliac fossa. Sometimes the

---

* Lloyd (Jordan). "Appendicitis and perityphlitis."—*Birmingham Medical Review*, Feb., 1894.

† Senn (Nicholas). "Appendicitis obliterans."—*Journal of the American Medical Association*, March 24, 1894. Abstract, *International Med. Mag.*, June, 1894.

‡ Edebohls (George M.). "Diagnostic palpation of the vermiform appendix."—*American Journal of Medical Sciences*, May, 1894, p. 487-492.

§ Bull (W. T.). "Appendicitis."—*Medical Annual*, 1894, p. 89.

pain may be in the latter situation from the commencement. Vomiting may be limited to a single occurrence, or may be continued with greater or less frequency. There is at times constipation, but often there is diarrhea. Generally there is tenderness in the right iliac fossa, but the exact point is by no means constant. There may be simply a sense of resistance on deep pressure, or distinct tumefaction may be noted. Fowler also shows that the fact that the pain is so seldom located about the cecum in the commencement of the disease, is in great measure responsible for many errors which have been made in diagnosis. In the great majority of cases it is at first referred to the region of the umbilicus and to the epigastrium.

Shrady* believes in the diagnostic importance of localized pain in the right iliac fossa, and has never met an instance in which it was absent. Pains in the epigastric, umbilical, hypochondriac and hypogastric regions he believes are mostly colicky and reflex. Circumscribed muscle tension in the right iliac fossa is an important sign, and occurs in about forty per cent. of the cases. Tumor rarely shows itself before the third day after the appearance of localized pain or tension, and not until agglutination or circumscribed effusions have occurred. Dullness on percussion is a comparatively late symptom, and when the abscess is behind, the cecum can rarely, if ever, be demonstrated. Fluctuation does not appear, as a rule, until the second week. Œdema of the overlying integument is a sure indication of paratyphlitic abscess. Vomiting is an early symptom. Appendicitis may be simulated by psoas abscess, morbus coxarius, renal and biliary colic, tubal and ovarian disease, intussusception, malignant tumors of colon or cecum, perinephritic abscess, typhoid fever, and even floating kidney; and in acute and grave cases, where sudden peritonitis appears, perforating ulcers of the stomach or intestine and rupture of the gall-bladder have been mistaken for perforation of the appendix. Rectal examination will sometimes aid in diagnosis, but in most cases it will not.

*Shrady (George F.). "Some points in the diagnosis of appendicitis."—*Medical Record*, January 6, 1894.

Gage* points out that the pain in these cases is often at first general and then local. It may be very severe or quite moderate in cases which at operation present very similar conditions. The location of the pain is of but little value, and "McBurney's point," as a diagnostic sign, should be discarded. Dulness is a sign of extremely limited value. Even the temperature may be normal, or nearly so, in most serious cases. Hemorrhagic pancreatitis has simulated appendicitis.

Jonas† records a remarkable case, occurring in a lady, where an enterolith gave rise to symptoms closely simulating those of appendicitis.

Rushmore,‡ in reviewing his experience, arrives at the following conclusions:—(1) Appendicitis is a surgical disease from its beginning; (2) its diagnosis is usually not difficult; (3) in doubtful cases exploratory laparotomy is justifiable; (4) appendicectomy, all things considered, offers the best chance, immediate and remote, for the patient; (5) the operation should be done at the earliest possible moment.

Fowler§ shows that the mode of termination of a case of appendicitis may be:—(1) By perforation prior to the formation of proper adhesions, thus giving rise to septic peritonitis; (2) infection of the peritoneum through the lymphatics or other channels of migration for the *bacillus coli communis* and other micro-organisms. Here the serous effusion may be—(*a*) encysted, and subsequently purulent; or (*b*) non-encysted, and occupy the general peritoneal cavity. In the case of the encysted variety a secondary rupture will lead to a rapidly fatal general septic peritonitis. (3) Rupture of an appendicital abscess and consequent peritonitis; (4) septic complications, arising from extension to and infection of the post-peritoneal connective tissue; (5) gangrenous inflammation of the appendix, of the adhesions which surround it, and of the neighbor-

*Gage (Homer). "Appendicitis: Some impressions derived from an experience of forty-four cases."—*Boston Medical and Surgical Journal*, May 24, 1894.

†Jonas (A. F.). "Enterolith mistaken first for appendicitis and then for carcinoma."—*Medical Record*, March 3, 1894.

‡Rushmore (John D.). "Some surgical thoughts on appendicitis from a clinical standpoint."—*Annals of Surgery*, May, 1894.

§Fowler (George R.). "Observations upon appendicitis."—*Annals of Surgery*, March, 1894, p. 330.

ing parts, including the blood-vessels, giving rise in the latter case, to fatal hemorrhage; (6) pylephlebitis, hepatic abscesses, with their sequelæ (purulent pleuritis, from extension, pneumonia, etc.); (7) the purulent accumulation may open into the cecum, rectum, or bladder, and the patient perish from septic troubles.

He also mentions the following among the conditions which may be associated with appendicitis:—Iliac phlebitis, iliac thrombosis, and hemorrhage; hepatic abscess; purulent pleuritis; purulent pericarditis; pelvic, lumbar, and parietal phlegmon; fecal or urinary communications.

Abbe* has met with intestinal obstruction occurring twelve days after operation for appendicitis, where the small intestine was adherent at the site of the appendix.

Gerster† has also met with intestinal obstruction following operation for the relief of gangrenous appendicitis.

The Keiths,‡ while admitting that appendicitis has become of the very greatest importance in abdominal surgery, believe "that, as with every new thing, the pendulum has swung too far towards operation, and that the time is coming, as it has already come in the operation for the removal of the ovaries, when a definite stand must be made against the indiscriminate removal of the appendix vermiformis." They stated that "one firm of instrument-makers in New York is reported to have made to order six dozen trusses for ventral hernia, following the removal of the appendix, and during the first six months of 1893!"

Burrell§ discusses many surgical points in the management of cases of appendicitis.

---

* Abbe (Robert). "Appendicitis complicated with intestinal obstruction."—*Annals of Surgery*, June, 1894, p. 677.

† Gerster (Arpad G.). "Excision of gangrenous appendix, followed by intestinal obstruction."—*Annals of Surgery*, June, 1894, p. 682.

‡ Keith (Skene) and Keith (George). "Text-Book of Abdominal Surgery, 1894, pp. 205-229.

§ Burrell (Herbert L.). "The after treatment of operations for appendicitis."—*Boston Medical and Surgical Journal*, May 3, 1894.

